# Mitochondrial Bioenergetics in Different Pathophysiological Conditions 2.0

**DOI:** 10.3390/ijms23105552

**Published:** 2022-05-16

**Authors:** Daniela Valenti, Anna Atlante

**Affiliations:** Institute of Biomembranes, Bioenergetics and Molecular Biotechnologies (IBIOM)-CNR, Via G. Amendola122/O, 70126 Bari, Italy

Mitochondria, traditionally identified as the powerhouses of eukaryotic cells, constitute a dynamic network of signaling platforms with multifaceted key roles in cell metabolism, proliferation and survival [1]. Dysfunctional mitochondria are critically involved in the pathogenesis of a wide range of metabolic, neurodegenerative, immune and neoplastic disorders [2,3].

In the second volume of this Special Issue, three review papers and twelve original articles were published dealing with compelling topics related to mitochondrial bioenergetics and other related multifunctional roles of mitochondria in various pathophysiological contexts.

Oxidative phosphorylation (OXPHOS) is the primary bioenergetic function of mitochondria, although the landscape of mitochondrial functions is continuously growing to include more aspects of cellular homeostasis. Thanks to the application of omics technologies to the study of the OXPHOS apparatus, novel features have emerged from the classification of new proteins identified as mitochondrial, thus adding details to the mitochondrial proteome and defining novel metabolic cellular interactions, especially in the human brain. In this context, Zafardino and coworkers tackled the dysfunction of mitochondrial bioenergetics in the brain, focusing on different aspects of mitochondrial structure, function and dysfunction by reviewing several genetic defects, mostly involving the nervous tissue, and briefly discussing the future directions of the multi-omics study of human brain disorders [4].

Mitochondria attracted renewed attention from the scientific community due to the recent discovery of their intercellular translocation ability that can involve whole mitochondria, the mitochondrial genome or other mitochondrial components. The intercellular transport of mitochondria can occur in mammalian cells both in vitro and in vivo and in physiological and pathological conditions [5]. Mitochondrial transfer can provide an exogenous mitochondrial source, replenishing dysfunctional mitochondria, thereby improving mitochondrial faults or, as in in the case of tumor cells, changing their functional skills and response to chemotherapy. The review of Valenti et al. provided a synopsis of the state of knowledge on the intercellular trafficking of mitochondria by discussing its biological relevance, mode and the mechanisms underlying the process and its involvement in different pathophysiological contexts, highlighting its therapeutic potential for diseases with mitochondrial dysfunction primarily involved in their pathogenesis [5].

Mitochondrial dysfunction has been widely described in Alzheimer’s Disease (AD), the most common neurodegenerative disorder, the etiopathology of which still remains largely unclear due to its highly complex and multifactorial nature. Patro et al. discussed the up-to-date knowledge on the critical role of dysregulated ATP synthase and other components of mammalian mitochondrial bioenergetics as an early event in AD [6]. The authors debated whether such dysregulation could act as a trigger for the dysfunction of the organelle, which is a clear component in the etiopathology of AD. Consequently, the pharmacological modulation of the ATP synthase could be a potential strategy to prevent mitochondrial dysfunction in AD [6].

Several pediatric mitochondrial disorders, including Leigh syndrome (LS), influence mitochondrial genetics, development and metabolism, leading to complex pathologies and energy failure. To better identify LS severity, Bakare et al. calculated a glycolytic bioenergetics health index (BHI) for measuring mitochondrial dysfunction in LS patient fibroblast cells harboring varying percentages of pathogenic mutant mtDNA exhibiting deficiency in mitochondrial respiratory complex V or complex I [7]. The levels of the defective enzyme activities of the electron transport chain correlated with the percentage of pathogenic mtDNA. Subsequent bioenergetic assays showed that cell lines relied on both OXPHOS and glycolysis to meet energy requirements. Overall, these results suggest that a multi-pronged approach that takes into consideration the specific pathogenic mtDNA variant, along with a composite BHI ratio, can provide valuable support in better diagnosing and understanding the factors influencing disease severity and rapid fatality in LS [7].

The mitigation of the calcium-dependent destruction of skeletal muscle mitochondria is considered to be a promising adjunctive therapy in Duchenne muscular dystrophy (DMD). The study by Dubinin et al. investigated the effect of the intraperitoneal administration of alisporivir, a nonimmunosuppressive inhibitor of the calcium-dependent mitochondrial permeability transition (MPT) pore, on the state of skeletal muscles and the functioning of mitochondria in dystrophin-deficient mdx mice [8]. The authors showed that treatment with alisporivir reduces inflammation, thus improving muscle function in mdx mice. These effects of alisporivir were associated with recovery in the ultrastructural alterations of mitochondria; the normalization of respiration and oxidative phosphorylation and a decrease in lipid peroxidation, due to suppression of MPT pore opening; and an improvement in calcium homeostasis [8]. The MPT pore targeting approach may be used as an effective adjunctive strategy in the treatment of DMD.

Glioblastoma represents the highest grade and the most aggressive form of brain tumor [9]. Despite maximal resection surgery associated with radiotherapy and concomitant followed by adjuvant chemotherapy with temozolomide (TMZ), patients have a very poor prognosis due to the rapid recurrence and the acquisition of resistance to TMZ. In their study, Zampieri et al. explored the contribution of glioblastoma cell metabolism to TMZ resistance, finding that fitter mitochondria in TMZ-resistant glioblastoma cells are a direct cause of chemoresistance that can be targeted by inhibiting oxidative phosphorylation and/or autophagy/mitophagy [10]. Unexpectedly, the authors found that the PARP inhibitor olaparib is also a mitochondrial Complex I inhibitor. They proposed that the anticancer activities of olaparib in glioblastoma and other cancer types combine DNA repair inhibition and the impairment of cancer cell respiration [10].

Mitochondrial dysfunctions are implicated in several pathologies, such as metabolic, cardiovascular, respiratory and neurological diseases, as well as in cancer and aging [2,3]. These metabolic alterations are usually evaluated in human or murine samples by mitochondrial respiratory chain enzymatic assays, by measuring the oxygen consumption of intact mitochondria isolated from tissues or from cells obtained after physical or enzymatic disruption of the tissues. However, these methodologies do not maintain tissue multicellular organization and cell–cell interactions, known to influence mitochondrial metabolism. The study by Kluza et al. set up and validated a new strategy to optimally assess mitochondrial function in murine tissues samples using the XF24 Extracellular Flux Analyzer (Seahorse) and discussed the advantages and limitations of this technological approach [11].

Studies on mitochondrial bioenergetics in different pathophysiological conditions not only aim at understanding the changes in the molecular mechanisms of energy-transducing membranes, leading to pathology, but also aim at developing novel pharmaceuticals capable of mitigating and/or reversing the pathological changes. In their study, Gasanoff et al. examined the effects of melittin, a bee venom membrane-active peptide, on mitochondrial respiration and the cell viability of healthy human lymphocytes (HHL) and Jurkat cells, as well as on lymphoblasts from acute human T cell leukemia [12]. The results of their analysis show that melittin is more cytotoxic to human lymphoblasts derived from acute T cell leukemia than to healthy human lymphocytes. This study on model membranes suggests that melittin penetrates the plasma membrane of lymphoblastic leukemia cells with a higher proficiency than that of the plasma membranes of healthy lymphocytes [12]. The novel findings of this paper encourage the potential exploration of melittin use, not only as an anti-cancer drug, but also as a novel potential pharmaceutical agent to treat the pathologies of mitochondrial bioenergetics not related to oncological conditions.

Mutations in either mitochondrial DNA (mtDNA) or nuclear genes that encode mitochondrial proteins may lead to dysfunctional mitochondria, giving rise to mitochondrial diseases. Interestingly, the methylation of mtDNA has been associated with various clinical pathologies. The study of Mposhi et al. set out to assess whether mtDNA methylation could explain impaired mitochondrial function in patients diagnosed with myopathy without known underlying genetic mutations [13]. The authors of this explorative study provided indications that mtDNA methylation in the CYTB gene was elevated in the muscle tissue of 14 myopathy patients when compared to healthy controls and that this parameter correlated with ATP production [13].

Mitochondrial functional integrity depends on protein and lipid homeostasis in the mitochondrial membranes and disturbances in their accumulation can cause disease. AGK, a mitochondrial acylglycerol kinase, is not only involved in lipid signaling but is also a component of the TIM22 complex in the inner mitochondrial membrane, which mediates the import of a subset of membrane proteins. AGK mutations can alter both phospholipid metabolism and mitochondrial protein biogenesis, contributing to the pathogenesis of Sengers syndrome, a rare autosomal recessive disorder caused by mutations in the *AGK* gene and characterized by hypertrophic cardiomyopathy, congenital cataracts and mitochondrial myopathy [14]. The article by Barbosa-Gouveia and coworkers described the case of an infant carrying a novel homozygous AGK variant, who was born with congenital cataracts, critical congenital dilated myocardiopathy, hyperlactacidemia and died 20 h after birth [15]. A decrease of 96-bp in the length of the AGK cDNA sequence was detected. The homozygous variant was shown to exert a molecular impact on the integrity of the mitochondrial respiration system in the patient’s fibroblasts, causing an imbalance in the mitochondrial metabolites and leading to secondary dysfunction of OXPHOS [15].

DNA damage and mitochondrial dysfunction are defining characteristics of aged vascular smooth muscle cells (VSMCs) found in atherosclerosis. Pink1 kinase regulates mitochondrial homeostasis and recycles dysfunctional organelles critical for maintaining energetic homeostasis. In their study, Docherty et al. generated a new vascular-specific Pink1 knockout and assessed its effect on VSMC-dependent atherogenesis in vivo and VSMC energetic metabolism in vitro [16]. During atherogenesis, the authors found that Pink1 knockout affects the development of plaque quality rather than plaque quantity by decreasing VSMC and extracellular matrix components, collagen and elastin [16]. Pink1 protein was found to play a critical role in the wild-type VSMC response to metabolic stress by inducing a compensatory increase in mitochondrial hexokinase II, which catalyzing the first irreversible step in glycolysis. The authors suggested that Pink1 might tether a pool of mitochondrial hexokinase II to ensure that a shift toward glycolysis can effectively occur when mitochondrial respiration results are compromised [16].

The cardiovascular disease of atherosclerosis is characterized by aged vascular smooth muscle cells and compromised cell survival. Analysis of human and murine plaques detects markers of DNA damage, such as p53, Ataxia telangiectasia mutated (ATM) and defects in mitochondrial oxidative metabolism as major indicators. Comparing wild-type VSMCs from an ApoE model of atherosclerosis with Pink1 knockout of inducible mitochondrial dysfunction, Docherty et al. showed WT Pink1 to be essential for normal cell viability, while the antiaging protein Klotho has been shown to perform an important role in VSMC bioenergetics, requiring Pink1 to mediate energetic switching between oxidative and glycolytic metabolism [17]. Klotho improved the VSMC phenotype and, if targeted at the plaque early in the disease, could be a useful strategy to delay the effects of plaque ageing and improve VSMC survival.

Pallag et al. investigated the effect of mitochondrial proline oxidation in providing sufficient bioenergetic drive for supporting mitochondrial ATP production when respiratory Complex I (CI) was inhibited [18]. The authors found that the oxidation of proline inside mitochondria isolated from various mouse tissue leads to the transfer of electrons to ubiquinone in mitochondria that express proline dehydrogenase (ProDH). In CI-inhibited mouse liver and kidney mitochondria that exhibited high levels of proline oxidation and ProDH activity, proline catabolism generated a sufficiently high membrane potential to be able to maintain ATP production by the mitochondrial F1FOATPase [18]. The results suggest that proline catabolism can bypass a CI blockade, thus preventing bioenergetic collapse.

The study by Lay et al. analyzed the cytopathological outcomes of knocking down the expression of mitochondrial Complex II subunits in the simple model organism of *Dictyostelium discoideum* [19]. The mitochondrial Complex II is composed of four core subunits and mutations to any of the subunits result in lowered Complex II activity. Surprisingly, although mutations in any of the subunits can yield similar clinical outcomes, there are distinct differences in the patterns of clinical disease most commonly associated with mutations in different subunits. The study deployed antisense inhibition to individually knock down the expression of three of the Sdh subunits (SdhA, B and C), showing how the partial loss of SdhA resulted in mitochondrial dysfunction and AMPK-mediated phenotypic outcomes, whereas knocking down the expression of SdhB or SdhC subunits exhibited defects in growth on bacterial lawns and unique effects on mitochondrial respiration [19].

Experimental evolution with *Drosophila melanogaster* has been widely used for studying aging and longevity. The comparative study of Phillips et al. incorporated metabolomic data into DNA and RNA sequencing framework to gain deeper insights into the physiological and genetic mechanisms underlying longevity differences in three groups of experimentally evolved *Drosophila melanogaster* populations with different aging and longevity patterns [20]. Combining genomic and metabolomic data, the authors provided a list of biologically relevant candidate genes, among which was found significant enrichment for genes and pathways associated with neurological development and function and carbohydrate metabolism [20]. Neurological dysregulation and carbohydrate metabolism are known to be associated with accelerated aging and reduced longevity. More broadly, these findings demonstrate the value of combining multiple types of omic data with experimental evolution when attempting to dissect the mechanisms underlying complex and highly polygenic behaviors, such as aging.

## Data Availability

Not applicable.
